# Rules of Connectivity-Dependent Phonon Interference in Molecular Junctions

**DOI:** 10.1021/acs.nanolett.5c00225

**Published:** 2025-04-08

**Authors:** Liyuan Zheng, Erfan Norouzi Farahani, Abdalghani H. S. Daaoub, Sara Sangtarash, Hatef Sadeghi

**Affiliations:** Quantum Device Modelling Group, School of Engineering, University of Warwick, CV4 7AL Coventry, United Kingdom

**Keywords:** single-molecule junction, thermal conductance, phonon transport, thermoelectricity, phonon interference

## Abstract

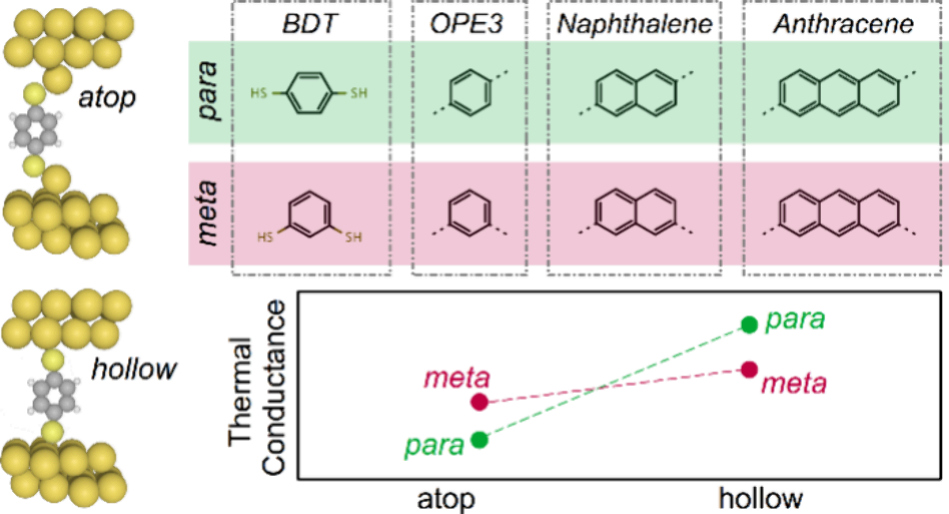

Controlling nanoscale heat flow is crucial for advanced electronics. Phonons, the primary heat carriers in molecules, exhibit wave-like behavior and can interfere, leading to phonon interference (PI). This study reveals that PI in molecular junctions connected to gold electrodes through different contact points deviates significantly from electron quantum interference (QI). Contrary to QI, *meta*-connected benzenedithiol (BDT), oligophenylene ethynylene (OPE3), bis(phenylethynyl)naphthalene, and bis(phenylethynyl)anthracene junctions can exhibit higher thermal conductance than *para*-connected ones. This arises from multiple phonon transmission channels and long-range interatomic interactions, both absent in electronic systems. Single-channel phonon transport shows an inverted interference pattern compared to electrons, while multichannel transport resembles QI. It is also demonstrated that dephasing effects have minimal effects on the PI at this scale. Our work provides key insights into phonon transport and offers design strategies for manipulating thermal conductance in molecular junctions, with implications for thermoelectric devices and nanoscale thermal management.

The study of phonon transport in molecules is a rapidly developing field with a broad range of applications.^1,2^ Phonons are the collective excitations of atoms in a solid or molecule, and they play a crucial role in determining the thermal conductance of materials.^3,4^ In recent years, there has been growing interest in understanding how phonons transmit through nanoscale materials and in particular molecular systems, because this can be used to design new materials with engineered thermal properties for thermoelectric energy harvesting, solid state refrigeration and on-chip cooling, or thermal management.^1,2,4^ Since phonons are the main carrier of heat in molecules,^5^ understanding their transmission through molecules is important to develop design strategies to engineer and control heat transport.

Phonons can exhibit wave-like behavior, just like light waves. As such, they can potentially interfere when they propagate through molecules, leading to phonon interference (PI) patterns that influence how heat is conducted. This opens up exciting possibilities for manipulating heat flow at the nanoscale. Studies have shown that the transport of heat through molecular junctions can be influenced by their anchoring group to electrodes,^6^ their internal phonon modes,^7−9^ stacking,^10−13^ their functionalization with heteroatoms or pendant groups,^7,14,15^ and their connectivity to electrodes.^14,16,17^ By understanding and controlling PI, we can potentially design materials with specific thermal conductances, leading to highly efficient thermoelectric devices or thermal insulators that prevent heat loss.^1,2,18−20^ While there have been few measurements of thermal conductance through single molecules,^5,21,22^ such thermal conductance measurement has proven to be difficult, so it is crucial to develop novel strategies to control and engineer phonon transport through molecules, enabling more focused synthesis and measurement of the most promising molecular structures.

In this paper, we aim to explore phonon transport in molecules connected to electrodes from different connection points. Such junctions are expected to exhibit interference effects because phonons being transmitted through the network of atoms can interfere at nodes, resulting in either destructive or constructive PI, depending on their transmission path. While recent investigations of PI in molecules suggest similar interference behavior for phonons and electrons,^17^ we demonstrate that rules governing PI in molecules differ substantially from those of electrons. We discuss the reasons for this discrepancy and present design strategies to engineer and control PI in molecular junctions. Our investigation employed a range of theoretical and computational methods, including density functional theory, phase-coherent phonon transport calculations, and non-equilibrium heat transport simulations. These methods enabled us to understand the underlying physics, develop new design principles, and investigate the factors influencing phonon transport behavior.

Figure 1a–d shows the structures of benzenedithiol (BDT), oligophenylene ethynylene (OPE3), bis(phenylethynyl)naphthalene (Np), and bis(phenylethynyl)anthracene (Anth) molecules studied here, with *para* (*p*) and *meta* (*m*) connections to two gold electrodes. We first perform geometry optimization of the molecules in the gas phase and calculate their phonon frequencies and vibrational modes using two implementations of density functional theory: SIESTA^23^ and Gaussian g16.^24^ The molecules are then placed between two gold electrodes, and their ground state geometries and forces are calculated. These are used to construct dynamical matrices. We then combine these dynamical matrices with our transport code, GOLLUM,^25−27^ to calculate phonon transmission *T*_p_ and thermal conductance *κ*_p_ through each junction. Later, we also employ molecular dynamics simulations using the LAMMPS code^28,29^ and calculate *κ*_p_ using both equilibrium and non-equilibrium heat transport methods. See the details of the methods in the Supporting Information.

**Figure 1:**
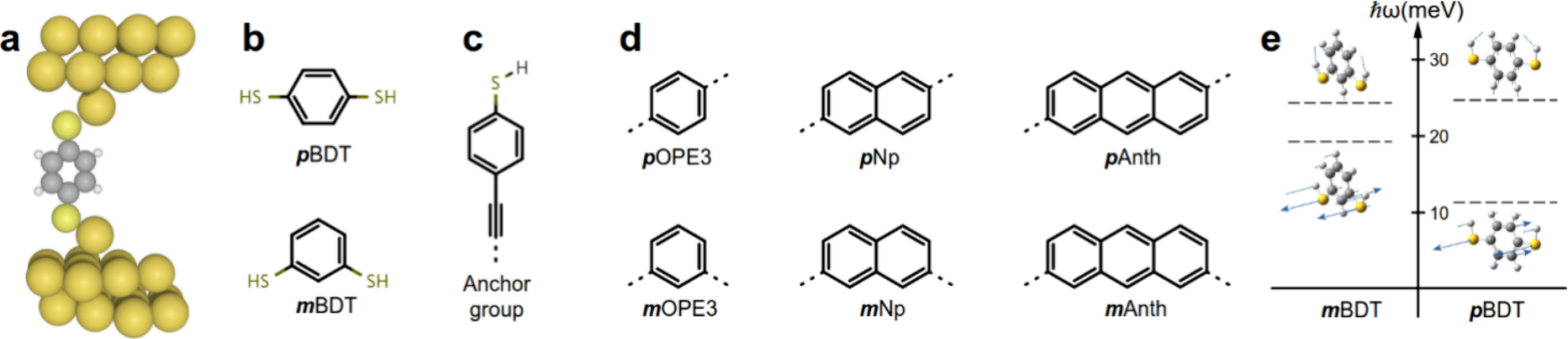
Molecular structure of junctions. (a) Example of a junction studied here, where a BDT molecule is connected to two gold electrodes through *para* connections and atop binding configurations. (b) Molecular structure of *meta* (*m*) and *para* (*p*) BDTs. (c) Anchor group used to form junctions with (d) benzene (OPE3), naphthalene (Np), and anthracene (Anth) molecular cores. (e) Phonon energy level diagram for a BDT molecule within the energy range relevant for gold electrodes. The Debye frequency of gold electrodes is around 21 meV, meaning that phonons with energies below this value can be transmitted through the electrodes.

Figure 2 shows *T*_p_ and the corresponding *κ*_p_ of BDTs with *meta* and *para* connections to electrodes (Figure 2a) with two different sulfur–gold binding configurations. Figure 2b shows the *T*_p_ of *m*BDT and *p*BDT connected to electrodes with a tip, where sulfur anchor groups are connected to one gold atom on the tip (atop binding). Figure 2c shows similar transmissions but with molecules connected to electrodes through binding between the sulfur atom in the anchor and three gold atoms in the flat surface of the electrodes (hollow binding). The corresponding *κ*_p_ values are shown in Figure 2d.

**Figure 2:**
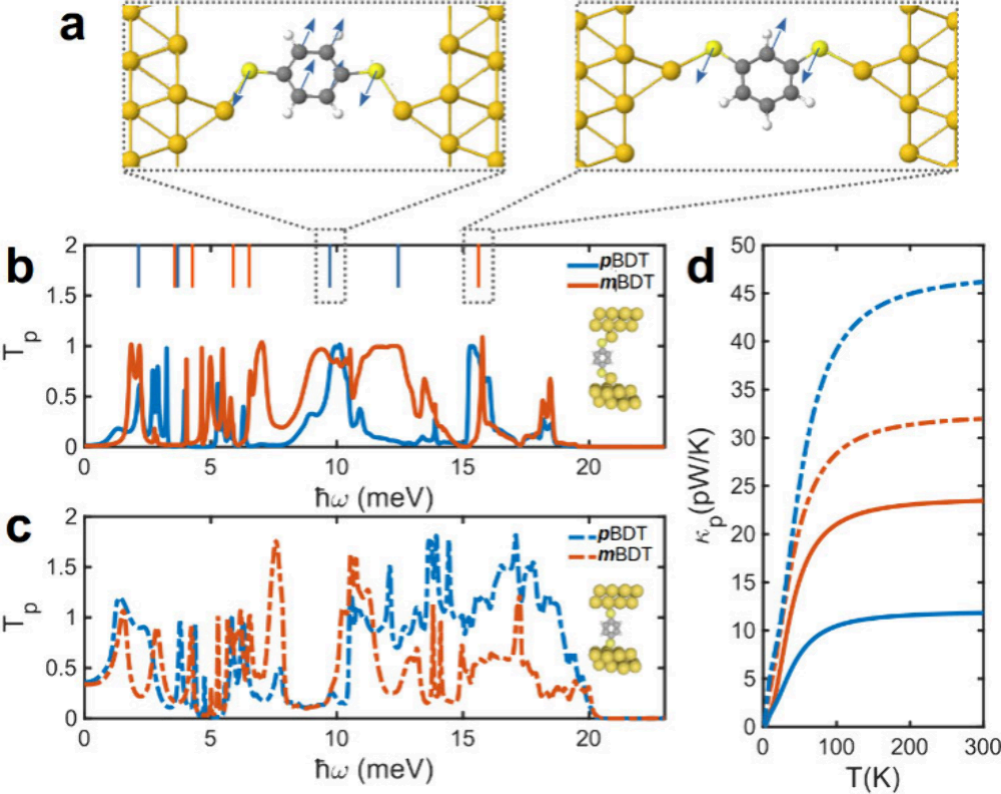
DFT phonon transport through the BDT molecules connected to gold electrodes through *para* and *meta* configurations. (a) Example of vibrational modes. Phonon transmission coefficients for *m*BDT and *p*BDT through a junction with (b) atop binding and (c) hollow binding to electrodes. (d) Phonon thermal conductance. Solid and dashed lines represent atop and hollow binding to electrodes, respectively.

Interestingly, the *κ*_p_ of *m*BDT connected through atop binding is higher than that of *p*BDT (Figure 2d). This contradicts expectations based on quantum interference (QI) of electrons in BDTs because studies show that *p*BDT exhibits higher electron transmission and electrical conductance than *m*BDT due to QI effects.^30^ This contrasting behavior of PI and QI in BDTs connected to electrodes through the atop configuration suggests that the rules governing PI differ from those of QI. We also examined the *κ*_p_ values of BDTs connected to electrodes through hollow binding between sulfur and gold and found that *p*BDT exhibits a higher *κ*_p_ than *m*BDT (Figure 2d).

In what follows, we demonstrate that this discrepancy is due to two main effects. First, unlike electrons, whose transport is dominated by one conduction channel (e.g., π orbitals in conjugated molecules like BDT), phonon transport through the molecule can occur via equally important multiple conduction channels (e.g., hollow binding). Second, long-range interatomic interactions lead to coupling between atoms beyond their first nearest neighbor in the molecule. Furthermore, the interchannel interference between different transport channels can also influence the PI pattern.

We first discuss the effect of multiple phonon transmission channels. For this, we partition the dynamical matrix into three parts for the *x*, *y*, and *z* channels to study *T*_p_ through the molecule due to individual phonon conduction channels (see Methods for details). This approach mirrors the study of electron transport through different orbitals, such as σ or π orbitals. Although QI in conjugated molecules is usually dominated by the π system, allowing established rules to predict behavior,^26^ these rules are weakened when contributions from other orbitals, such as σ orbitals, become comparable.^31,32^

Figure 3a–f illustrates the *T*_p_ for the *x*, *y*, and *z* channels, the total phonon transmission, and the corresponding *κ*_p_ for *m*BDT connected to electrodes through atop (Figure 3a) and hollow (Figure 3d) binding configurations, respectively. For the atop binding configuration, the *T*_p_ for individual channels is always below 1 across all phonon frequencies ω, indicating that only one channel contributes to transport at any given frequency (Figure 3b). Similarly, the total *T*_p_ (blue line in Figure 3b) remains below 1 for almost all frequency ranges, suggesting that a single conduction channel dominates phonon transmission. Notably, when the molecule is connected to electrodes via an atop configuration (Figure 3b,c), the *κ*_p_ through the *z* channel (the transport direction) is considerably higher than that of the *x* and *y* channels (transverse modes). This is due to significant anisotropy in Au–S coupling in different directions for the atop configuration, where the coupling is significantly greater along the transport direction than in the transverse directions. This leads to a larger width of phonon transport resonances for the *z* channel (Figure 3b) and, consequently, a larger contribution to the thermal conductance, which is proportional to the area under the *T*_p_ curve. This demonstrates that when phonon transport is dominated by a single transmission channel, the PI pattern is opposite to that of QI. To investigate how this behavior changes with multiple phonon transmission channels, we examined the *κ*_p_ of *m*BDT connected to electrodes via a hollow configuration (Figure 3d). Interestingly, the trend is reversed when the molecule connects to electrodes through more than one Au–S interaction, as depicted in panels e and f of Figure 3.

**Figure 3:**
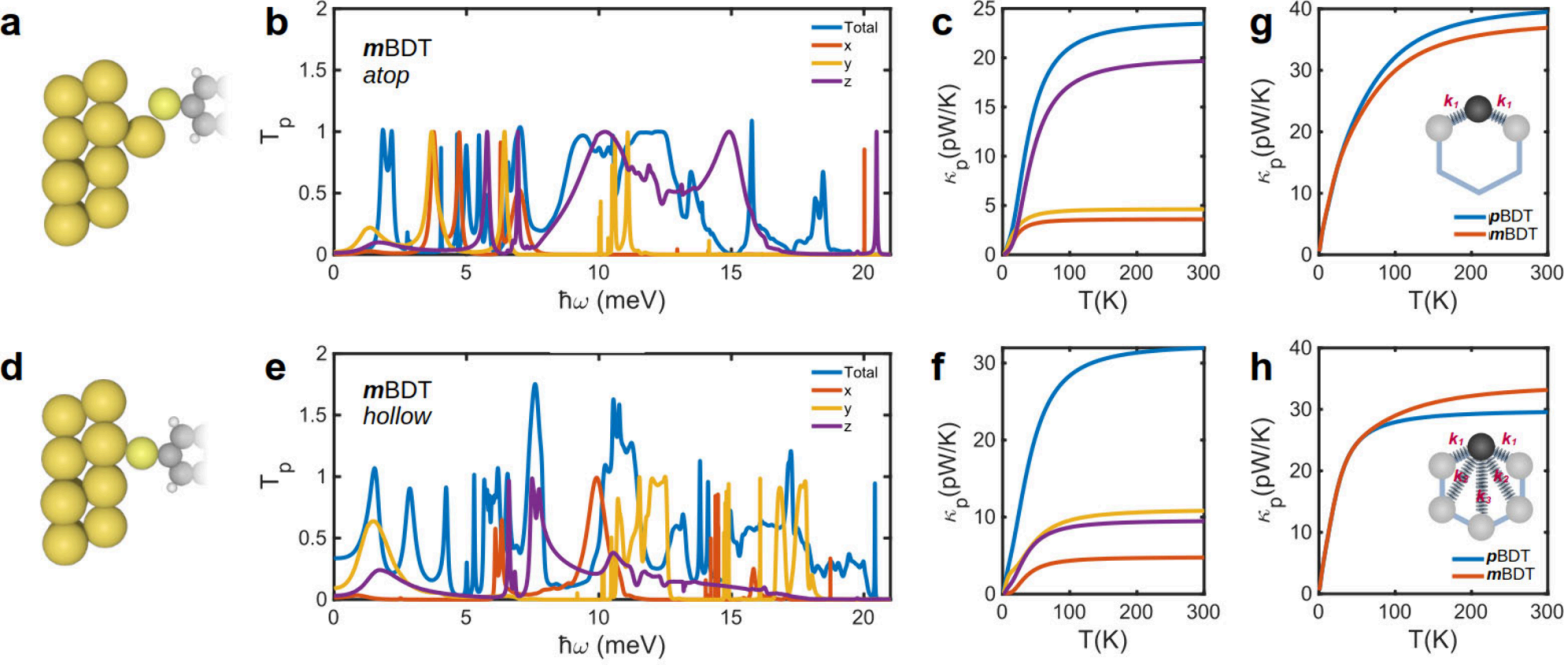
Underlying physics of PI. Molecular structures of junctions formed by (a) atop and (d) hollow binding to electrodes and (b and e) associated DFT phonon transmission and (c and f) DFT thermal conductance for the *x*, *y*, and *z* channels and the total transmissions and thermal conductances for *m*BDT. TB thermal conductance through benzene where atoms are coupled to (g) only their first nearest neighbor or (h) multiple atoms. The TB model in panels g and h is parametrized based on the dynamical matrix of isolated benzene molecules obtained using DFT Gaussian g16. The insets of panels g and h show examples of coupling between any atom and its nearest neighbor.

Figure 3e shows the *T*_p_ of *m*BDT through each individual channel for the hollow configuration. Evidently, the corresponding *κ*_p_ values (Figure 3f) are comparable across all channels. This is because Au–S coupling is more isotropic along different channels in hollow configurations, and therefore, all channels contribute to phonon transport. This highlights the significance of PI through equally important multiple phonon transmission channels. In such cases, the overall interference pattern resembles that of electrons. The *T*_p_ of *p*BDT through each individual channel for the atop and hollow configuration exhibits similar behavior to *m*BDT, with the *z* channel dominating phonon transport in the atop configuration unlike the hollow binding configuration, as shown in Figures S2 and S3. Notably, the sum of the *κ*_p_ values across individual channels often does not equal the total *κ*_p_. In the atop configuration of *p*BDT connected to electrodes (Figure S2c,e), the *κ*_p_ for a single channel even exceeds the total *κ*_p_. This indicates that while phonons are primarily injected through one channel when the molecule connects to an electrode via a single Au–S bond, strong coupling among the *x*, *y*, and *z* channels leads to strong hybridization and allows them to transmit through other channels within the molecule. This interchannel interference further suppresses *κ*_p_ and represents another notable difference between PI and QI in molecules.

It is worth mentioning that there is only one internal vibrational mode of BDT within the Debye frequency of the gold electrodes (Figure 1e). This mode is at approximately 19 and 11 meV for *m*BDT and *p*BDT, respectively. Based on this, one might expect only a single mode to contribute to transport. However, panels b and c of Figure 2 clearly show multiple resonances within the ∼21 meV range. To investigate this discrepancy, we analyzed the vibrational modes of the molecule between the electrodes. We found that the modes shown in Figure 2a (when the molecule is connected to electrodes) are similar to those in Figure 1e (gas phase molecule). Therefore, we concluded that *T*_p_ near these modes is due to the internal vibrational mode of BDT.

To further investigate the vibrational modes, we artificially increased the mass of the electrodes (Au atoms) to a large value. This shifted all modes associated with the Au atoms to very low frequencies, leaving only the modes of the BDT molecule between the electrodes (the levels shown in the inset of Figure 2b). In addition to the internal mode of BDT (Figure 1c), we found modes similar to the center-of-mass modes of BDT within the 21 meV range. These modes, which have zero frequency in gas phase BDT, shift to higher energies when the molecule is confined between electrodes (inset of Figure 2b). From this analysis, we concluded that the other resonances in *T*_p_ arise from the hybridization of the electrode surface modes with the internal modes of BDT. This highlights another remarkable difference between PI and QI. Furthermore, QI is typically discussed for electron energies between frontier orbitals, where often contributions from both frontier orbitals lead to a given QI pattern. However, this is not the case with phonons, as only a few modes contribute to transport.

Next, we investigated the effect of long-range interatomic interactions on *T*_p_ and *κ*_p_. We constructed a dynamical matrix using the Gaussian g16 code (see Methods) for the molecular core (benzene molecule) without any anchor groups. Analysis of the 36 × 36 dynamical matrix revealed significant interatomic interactions between not only each site and its first nearest neighbor but also the second and third nearest neighbors. This suggests that phonons can exhibit a different interference pattern compared to electrons. This difference arises because p orbitals on each atom are electronically coupled primarily to their first nearest neighbors, whereas interatomic interactions extend to more distant sites.

Guided by the 36 × 36 dynamical matrix, we parametrized a simpler 6 × 6 dynamical matrix (Table S1) and connected it to one-dimensional electrodes to examine the effect of second and third nearest-neighbor couplings on *T*_p_ and PI. Panels g and h of Figure 3 demonstrate how the presence or absence of this long-range coupling influences PI and consequently *κ*_p_. When such coupling exists, *m*BDT exhibits a higher *κ*_p_ than *p*BDT (Figure 3h). In contrast, the trend is reversed when only the first nearest-neighbor interactions are present (Figure 3g). The corresponding *T*_p_ values are shown in Figure S4. This simple model elucidates the underlying physics of connectivity-dependent PI and demonstrates that long-range interatomic interactions play a significant role in determining the PI pattern. This is plausible because the long-range interatomic forces, arising from Coulombic interactions that govern PI, are fundamentally different from the orbital overlaps that dominate electron transport. This PI effect is most pronounced when only one phonon transmission channel is dominant (e.g., in the atop binding configuration). When additional phonon transmission channels contribute (e.g., in the hollow binding configuration), the overall thermal conductance becomes higher for *p*BDT than for *m*BDT. This is because each phonon channel contributes to transport differently due to differences in interatomic interactions between atoms for each channel. Consequently, *T*_p_, including the position, amplitude, and width of resonances, and its amplitude between resonances, differs for each channel, leading to a more complex PI pattern.

To demonstrate the generality of connectivity-dependent PI, we also studied the *κ*_p_ of OPE3 molecules as well as molecules with naphthalene and anthracene molecular cores with *meta* and *para* connections to electrodes (Figure 4a). Figure 4 shows the results for these molecules, which exhibits similar behavior to BDT. Specifically, the *κ*_p_ is higher for *m*-OPE3, Np, and Anth when a single Au–S bond is formed (atop configuration), and the trend is reversed when multiple Au–S bonds are formed (hollow configuration), as shown in Figure 4b. The corresponding phonon transmission functions for these junctions are shown in Figure S5. These results demonstrate the significant influence of electrode–molecule contact and long-range interatomic interactions on *T*_p_ that can be utilized to engineer PI. It is also worth noting that the changes in thermal conductance as a function of the junction lengths are connectivity dependent, especially for hollow binding (Figure 4b). For *meta*-connected junctions with hollow binding, thermal conductance increases slightly with length (from OPE3 to Anth, dashed red curves), while it decreases with length for *para*-connected junctions (dashed blue curves). In contrast, thermal conductance remains almost unchanged as a function of length for junctions with atop binding (small fluctuations in *para* connectivities).

**Figure 4:**
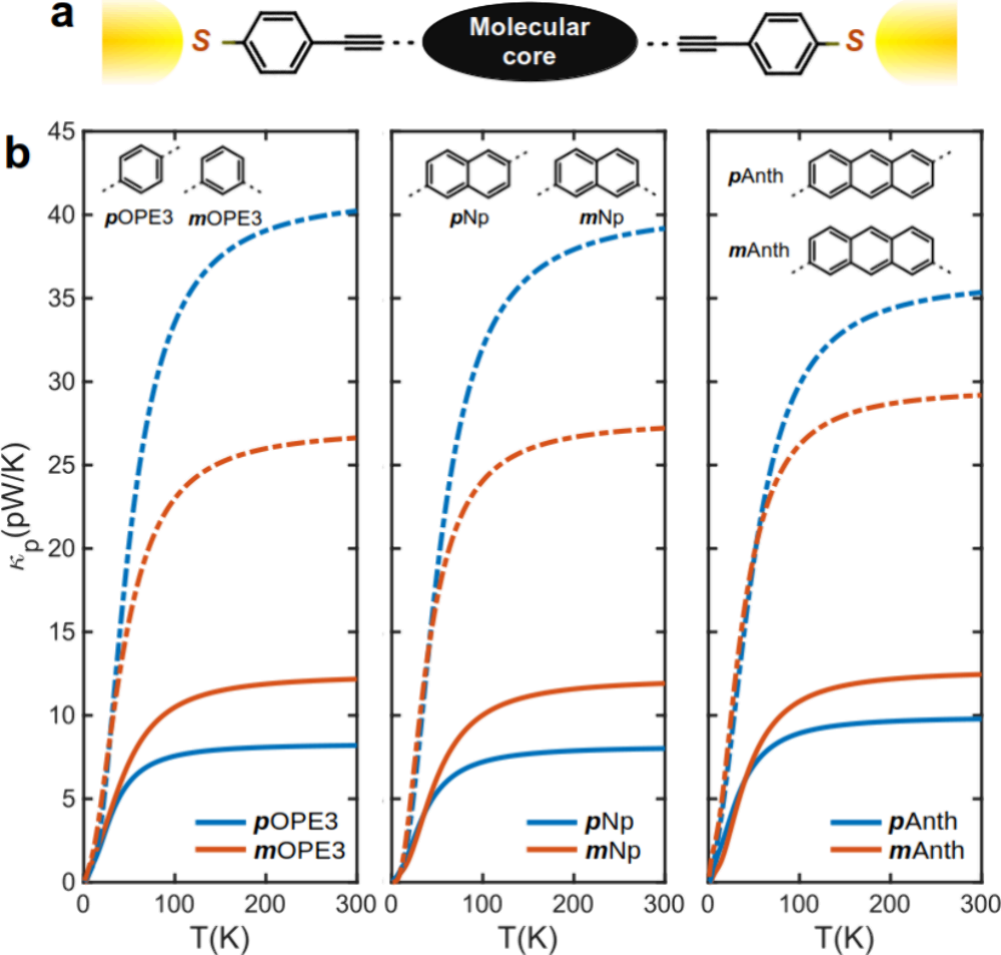
DFT thermal conductance for *p*- and *m*-OPE3, naphthalene, and anthracene junctions. (a) Schematic of a junction consisting of a molecular core (shown in the inset of panel b) and anchor groups connected to two gold electrodes. (b) Phonon thermal conductances of *p*OPE3 and *m*OPE3 (left), *p*Np and *m*Np (middle), and *p*Anth and *m*Anth (right) connected to gold electrodes with atop (solid lines) and hollow (dashed lines) binding to electrodes.

So far, we have used phase-coherent phonon transport to investigate PI in molecular junctions. To investigate the role of dephasing and non-equilibrium effects on PI, we used non-equilibrium molecular dynamics (NEMD) simulations (see Methods) to calculate *κ*_p_ through BDTs with different electrode connections and Au–S binding configurations. A constant external energy flow of 20 meV/ps was applied to the hot electrode (red in Figure 5a) and extracted from the cold electrode (blue in Figure 5a). This induced a temperature gradient in the metal electrodes, which we used to compute the *κ*_p_ values of the junctions (see Methods for details).

**Figure 5:**
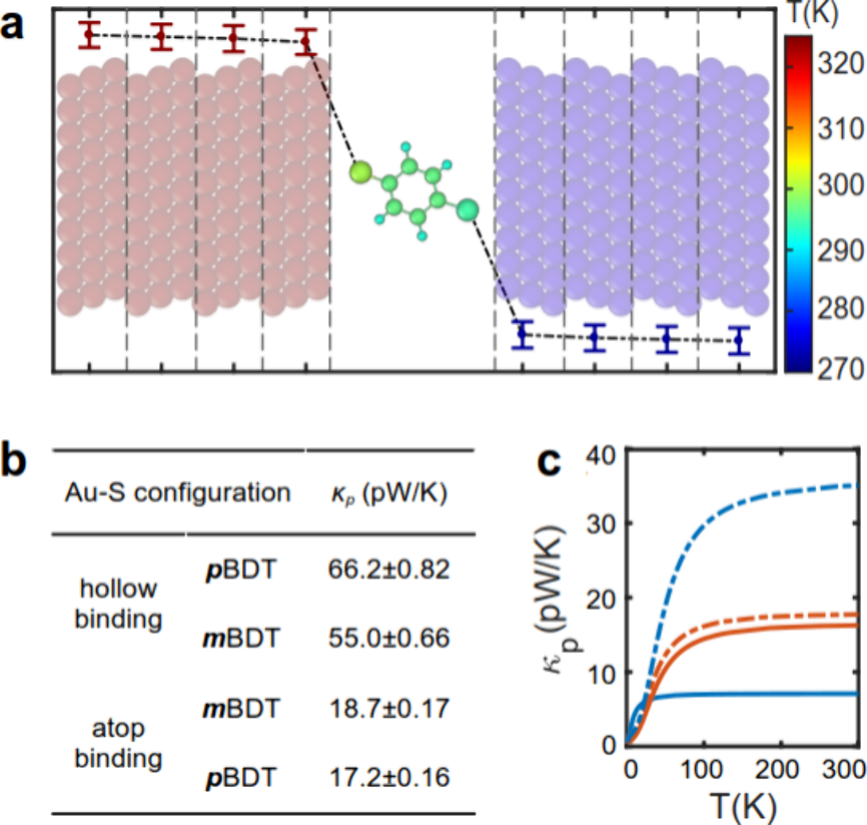
Non-equilibrium molecular dynamics (NEMD) thermal conductance for pBDT and mBDT junctions. (a) Temperature on each atom on the molecule and electrodes for a junction formed from *p*BDT connected to electrodes through a hollow Au–S configuration. The sizes of atoms in BDT are enlarged compared to their original size for the sake of clarity. The temperature profile is shown with a dashed line obtained by partitioning the electrodes into chunks as shown in panel a. (b) Summary of the NEMD results obtained for *κ*_p_ of *p*BDT and *m*BDT connected to electrodes through atop or hollow Au–S configurations and (c) *κ*_p_ of *p*BDT (blue) and *m*BDT (red) as a function of temperature using phase-coherent phonon transport calculations and force matrices obtained from MD simulations. Solid and dashed lines represent atop and hollow binding configurations, respectively.

Figure 5a shows the temperature at each atom in the molecule and at each chunk in the electrodes (group of atoms partitioned as shown in Figure 5a) for a junction formed by *p*BDT connected to the electrode through a hollow configuration. Temperature profiles for other junctions are shown in Figure S6. The main temperature decrease occurs at the electrode interfaces for all junctions (Figure 5a and Figure S6). Our NEMD results show that *m*BDT exhibits a higher *κ*_p_ than *p*BDT when connected to electrodes through the atop Au–S configuration (Figure 5b). This trend is reversed with the hollow Au–S configuration. These results agree well with our phase-coherent DFT transport calculations, demonstrating that dephasing effects play a minimal role in the *κ*_p_ values of small molecular junctions. Furthermore, we constructed a dynamical matrix from the force matrices obtained from the MD simulation and calculated *T*_p_ and *κ*_p_ using a method similar to that used for the DFT dynamical matrix (Figure S7). As shown in Figure 5c, the trends observed for the *κ*_p_ of BDTs are consistent across DFT results, NEMD calculations, and the MD-derived dynamical matrix. This suggests that dephasing and non-equilibrium effects do not distort PI, although they can lead to changes in the magnitude of *κ*_p_.

In summary, we investigated PI in molecular junctions formed by connecting molecules to gold electrodes through different contact points. Using first-principles calculations and molecular dynamics simulations, we demonstrated that PI in these systems deviates significantly from the established principles of QI observed for electrons. Contrary to QI, where *para*-connected junctions typically exhibit higher electrical conductance, we found that *para*-connected junctions can exhibit higher or lower phonon thermal conductance compared to *meta*-connected ones, depending on their contact configuration with electrodes. This unexpected behavior arises from the presence of multiple phonon transmission channels, long-range interatomic interactions within the molecule, and interchannel interference. When phonon transport is dominated by a single channel, the interference pattern is the inverse of that observed for electrons. However, when multiple channels contribute to transport, the overall interference pattern resembles QI. Furthermore, we showed that long-range interatomic interactions, a characteristic absent in electronic systems, significantly influence the PI pattern. This was elucidated through a simple tight-binding model and corroborated by first-principles simulations of BDT, OPE3, Np, and Anth molecules connected to gold electrodes in various configurations. These findings illuminate the complexities of phonon transport in molecular junctions, offering a strategy for controlling heat flow at the molecular level. These insights have significant implications for the design of next-generation thermoelectric devices and the optimization of thermal management in nanoscale electronics.

## Data Availability

The input files to reproduce simulation data can be accessed by contacting the authors.

## Supporting Information Available

Details of the computational methods and supporting figures, including phonon transmission and thermal condctances using DFT, TB, and MD simulations (PDF)
