# Supplementary material for: Rules of Connectivity-Dependent Phonon Interference in Molecular Junctions
Source: Nano Lett. 2025 Apr 8;25(16):6524–9. doi: 10.1021/acs.nanolett.5c00225 (PMC12023023; doi:10.1021/acs.nanolett.5c00225)
Supplement: Supplementary file 1 — nl5c00225_si_001.pdf [file nl5c00225_si_001.pdf]

Supporting Information

Rules of Connectivity-Dependent Phonon Interference in Molecular Junctions

Liyuan Zheng, Erfan Norouzi Farahani, Abdalghani H. S. Daaoub, Sara Sangtarash*, and Hatef Sadeghi*
Quantum Device Modelling Group, School of Engineering, University of Warwick, CV4 7AL Coventry, United Kingdom
* *sara.sangtarash@warwick.ac.uk* * *hatef.sadeghi@warwick.ac.uk*

Computational Methods

Density Functional Theory Calculations: The geometry of each structure studied in this paper was relaxed to the force tolerance of 10 *meV/Å* using the *SIESTA*[1] implementation of DFT, with a double-ζ polarized basis set (DZP) and the Generalized Gradient Approximation (GGA) functional with Perdew-Burke-Ernzerhof (PBE) parameterization. A real-space grid was defined with an equivalent energy cut-off of 300Ry. We also employed Gaussian 16 code[2] to calculate the vibrational modes, frequencies, and dynamical matrix of gas-phase molecules utilising the B3LYP functional and a basis set 6-311++g(d,p) with very tight convergence criteria.

Phonon Transport and Thermal Conductance: To calculate the phonon transport properties of the device[3,4], from the converged DFT calculation, a set of *xyz* coordinates were generated by displacing each atom from the relaxed *xyz* geometry in the positive and negative *x*, *y* and *z* directions with $\delta q' = 0.01Å$. The forces $F_i^q = (F_i^x, F_i^y, F_i^z)$ in three directions $q_i = (x_i, y_i, z_i)$ on each atom were then calculated and used to construct the dynamical matrix $D_{ij} = K_{ij}^{qq'}/M_{ij}$ where the mass matrix $M = \sqrt{M_i M_j}$ and $K_{ij}^{qq'} = \left[F_i^q(\delta q_j') - F_j^q(-\delta q_j')\right]/2\delta q_j'$ for $i \neq j$ obtained from finite differences. To satisfy momentum conservation, the K for $i = j$ (diagonal terms) is calculated from $K_{ii} = -\sum_{i\neq j} K_{ij}$. The phonon transmission $T_p(\omega)$ then is calculated using our quantum transport code, *GOLLUM*[5] from the relation $T_p(\omega) = Trace(\Gamma_L^p(\omega) G_p^R(\omega) \Gamma_R^p(\omega) G_p^{R\dagger}(\omega))$ where $\Gamma_{L,R}^p(\omega) = i(\sum_{L,R}^p(\omega) - \sum_{L,R}^{p\ \dagger}(\omega))$ describes the level broadening due to the coupling to the left L and right R electrodes, $\sum_{L,R}^p(\omega)$ are the retarded self-frequencies associated with this coupling and $G_p^R = (\omega^2 I - \mathrm{D} - \sum_L^p - \sum_R^p)^{-1}$ is the retarded Green's function, where *D* and *I* are the dynamical and the unit matrices, respectively. The phonon thermal conductance κ_p at temperature T is then calculated from $\kappa_p(T) = (2\pi)^{-1}\int_0^\infty \hbar\omega T_p(\omega)(\partial f_{BE}(\omega,T)/\partial T)d\omega$ where $f_{BE}(\omega,T) = (e^{\hbar\omega/k_B T} - 1)^{-1}$ is Bose–Einstein distribution function and $\hbar$ is reduced Planck's constant and k_B is Boltzmann's constant. To study phonon transmission and thermal conductance for different conduction channels, we partitioned D and used D^{xx}, D^{yy}, and D^{zz} to calculate transmission and corresponding thermal conductances through *x*, *y* and *z* channels, respectively.

Non-equilibrium Molecular Dynamics (NEMD) Simulations of Thermal Conductance: For the NEMD simulations, single BDT junctions was formed where the ***p***BDT and ***m***BDT are connected to two gold electrodes through either atop or hollow binding configurations. Each gold electrode was formed by 2160 Au(111) atoms, representing the bulk material. The last three gold layers at each end were fixed during the simulations to model the experimental junction gripping. Prior to the heat transport calculations, all geometries were relaxed using the Nose-Hoover thermostat and barostat[6,7] to set the temperature and pressure of the simulation box. A 1 *ns* MD simulation in the NPT ensemble was performed to apply vacuum pressure, followed by another 1 *ns* equilibrium simulation in the NVT ensemble to ensure room temperature conditions within the molecular junction.

The heat transport calculations were then performed using the NVE ensemble to control the energy of the atoms. An external energy flow (*J*) of 20 *meV/ps* was applied to the hot electrode and extracted from the cold electrode, inducing a temperature gradient across the junction. This gradient was used to compute the phonon thermal conductance of the junction as $\kappa_p = J/(T_h - T_c)$ where T_h and T_c are the temperatures of the hot and cold electrodes, respectively. The heat transport simulation was run for 22.5 *ns*, and the temperature values were recorded along the heat flow direction in both the molecule and the electrodes. To calculate the steady-state thermal conductance, the first 2.5 *ns* of the simulation were discarded. For each junction, we performed 20

independent runs and presented the resulting average values. This NEMD methodology has been previously validated for studying phonon heat transport in molecular junctions[8]. All MD calculations were performed using the LAMMPS MD software[9,10] with a 1 *fs* time step. Periodic boundary conditions were applied perpendicular to the heat transport direction to eliminate edge effects. The interatomic interactions in the gold electrodes and the organic molecule were described using the EAM[11] and GROMOS[12] force fields, respectively. The contact interaction between the sulphur anchor groups and the metal electrodes was modelled using an AMBER-based force field[13] for the atop configuration and an Au-S Morse potential[14] for the hollow configuration. Non-bonded interactions between atoms were modelled using the Lennard-Jones potential.

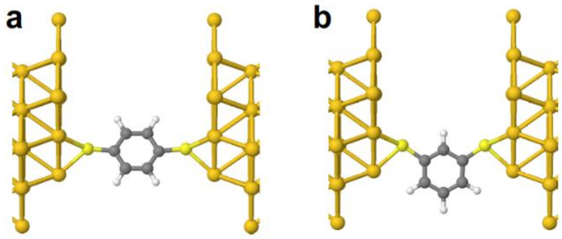

Figure S1. Molecular structure of BDTs connected to electrodes via hollow configuration.

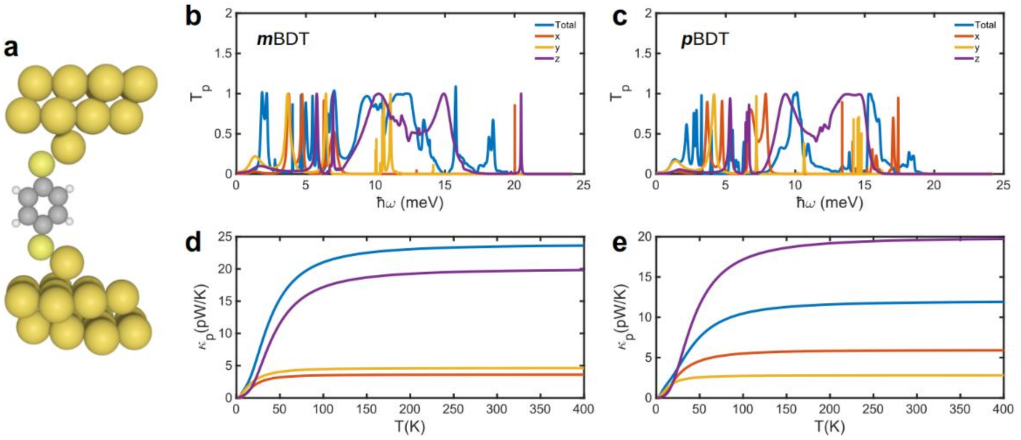

Figure S2. DFT Transmission and thermal conductance for ***p***BDT and ***m***BDT connected to electrodes through the atop binding configuration.

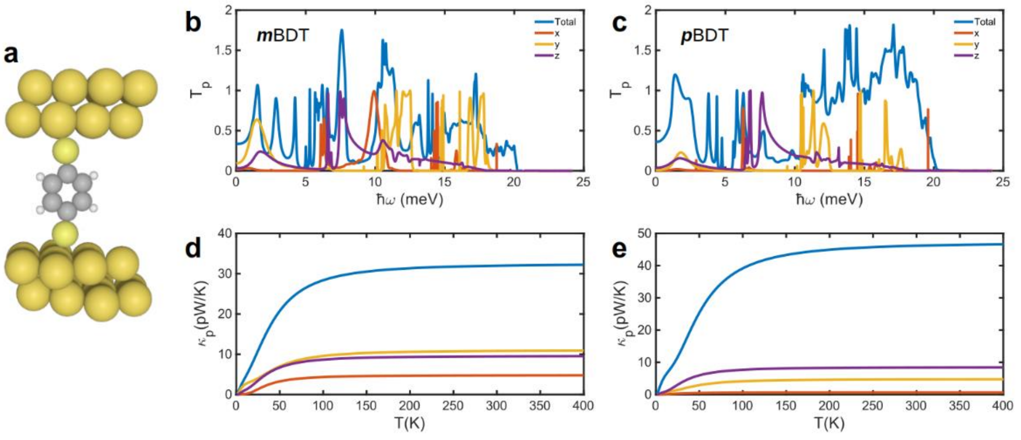

Figure S3. DFT Transmission and thermal conductance for ***p***BDT and ***m***BDT connected to electrodes through the hollow binding configuration.

Table S1. Simple 6x6 dynamical matrix D (eV/(Åu)) for a benzene ring with 6 sites. This molecular core is connected to 1D electrodes with spring constant -1 eV/(Åu) with molecule-electrode coupling -0.07 eV/(Åu). k_1, k_2 and k_3 are -0.05, -0.042, -0.033 eV/(Åu), respectively.

$$D = \begin{bmatrix} 0.217 & -0.050 & -0.042 & -0.033 & -0.042 & -0.050 \\ -0.050 & 0.217 & -0.050 & -0.042 & -0.033 & -0.042 \\ -0.042 & -0.050 & 0.217 & -0.050 & -0.042 & -0.033 \\ -0.033 & -0.042 & -0.050 & 0.217 & -0.050 & -0.042 \\ -0.042 & -0.033 & -0.042 & -0.050 & 0.217 & -0.050 \\ -0.050 & -0.042 & -0.033 & -0.042 & -0.050 & 0.217 \end{bmatrix}$$

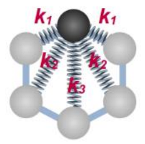

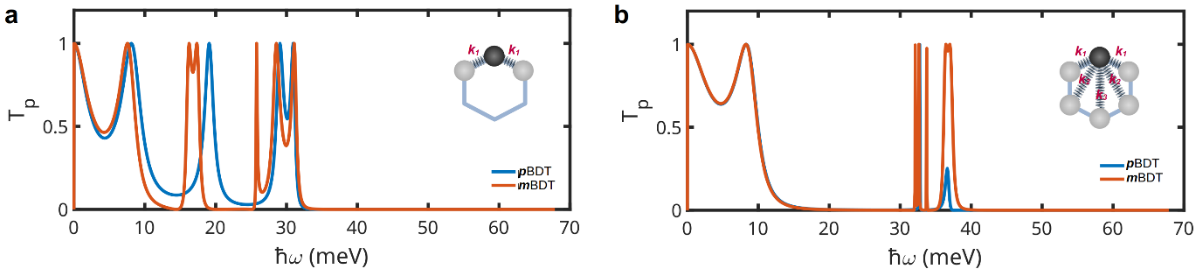

Figure S4. TB phonon transmission function for benzene with (a) only first nearest coupling, (b) 1st, 2nd, and 3rd nearest coupling connected to 1D electrodes.

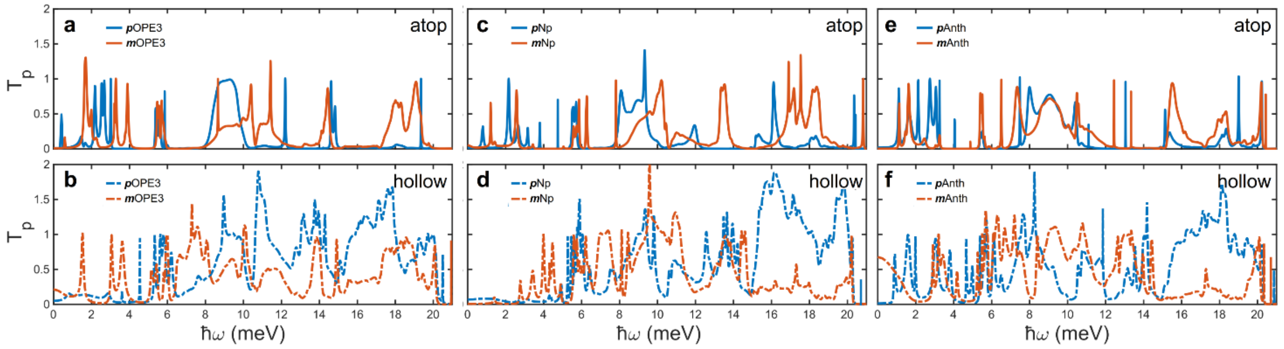

Figure S5. DFT phonon transmission functions for para- and meta- OPE3 (a,b), Naphthalene (c,d) and Anthracene (e,f) junctions. Top and bottom row figures represents atop and hollow binding configurations to electrodes, respectively.

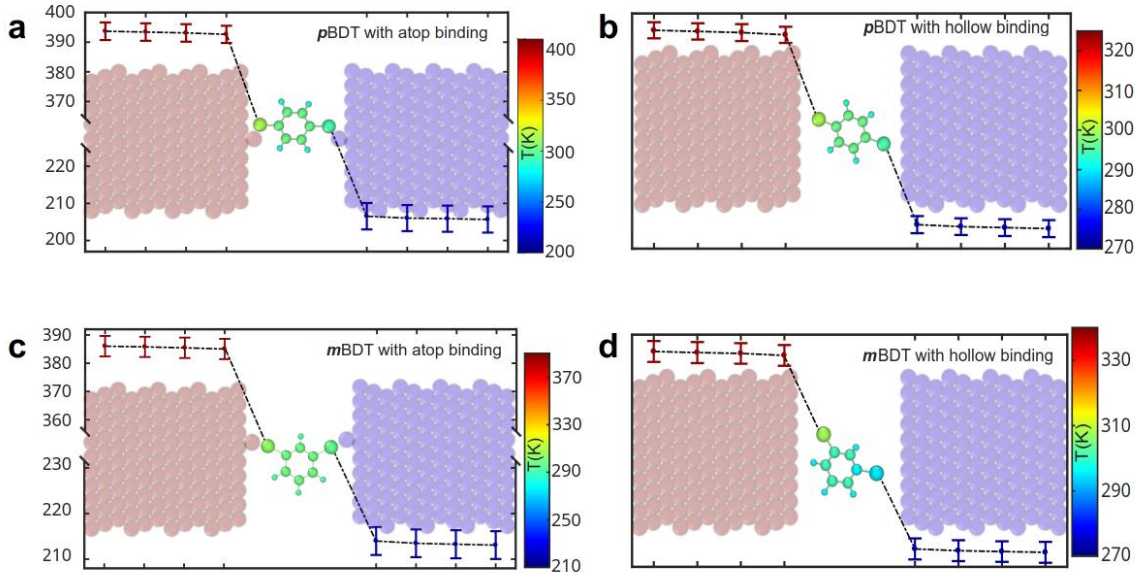

Figure S6. Temperature profiles for ***p***BDT and ***m***BDT binded to electrodes through (a,c) atop and (b,d) hollow configurations, respectively.

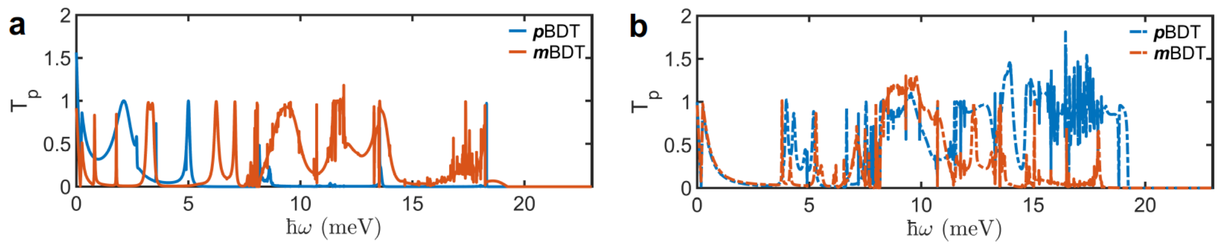

Figure S7. MD transmission functions for ***m***BDT and ***p***BDT through (a) atop and (b) hollow binding to electrodes.

References

(1) Soler, J. M.; Artacho, E.; Gale, J. D.; García, A.; Junquera, J.; Ordejón, P.; Sánchez-Portal, D. The SIESTA Method for Ab Initio Order- N Materials Simulation. *J. Phys. Condens. Matter* **2002**, *14* (11), 2745–2779. https://doi.org/10.1088/0953-8984/14/11/302.

(2) Frisch, M. J.; Trucks, G. W.; Schlegel, H. B.; Scuseria, G. E.; Robb, M. a.; Cheeseman, J. R.; Scalmani, G.; Barone, V.; Petersson, G. a.; Nakatsuji, H.; Li, X.; Caricato, M.; Marenich, a. V.; Bloino, J.; Janesko, B. G.; Gomperts, R.; Mennucci, B.; Hratchian, H. P.; Ortiz, J. V.; Izmaylov, a. F.; Sonnenberg, J. L.; Williams; Ding, F.; Lipparini, F.; Egidi, F.; Goings, J.; Peng, B.; Petrone, A.; Henderson, T.; Ranasinghe, D.; Zakrzewski, V. G.; Gao, J.; Rega, N.; Zheng, G.; Liang, W.; Hada, M.; Ehara, M.; Toyota, K.; Fukuda, R.; Hasegawa, J.; Ishida, M.; Nakajima, T.; Honda, Y.; Kitao, O.; Nakai, H.; Vreven, T.; Throssell, K.; Montgomery Jr., J. a.; Peralta, J. E.; Ogliaro, F.; Bearpark, M. J.; Heyd, J. J.; Brothers, E. N.; Kudin, K. N.; Staroverov, V. N.; Keith, T. a.; Kobayashi, R.; Normand, J.; Raghavachari, K.; Rendell, a. P.; Burant, J. C.; Iyengar, S. S.; Tomasi, J.; Cossi, M.; Millam, J. M.; Klene, M.; Adamo, C.; Cammi, R.; Ochterski, J. W.; Martin, R. L.; Morokuma, K.; Farkas, O.; Foresman, J. B.; Fox, D. J. Gaussian 16. 2016, p Gaussian 16, Revision C.01, Gaussian, Inc., Wallin.

(3) Sadeghi, H. Theory of Electron, Phonon and Spin Transport in Nanoscale Quantum Devices. *Nanotechnology* **2018**, *29* (37), 373001. https://doi.org/10.1088/1361-6528/aace21.

(4) Sadeghi, H.; Sangtarash, S.; Lambert, C. J. Oligoyne Molecular Junctions for Efficient Room Temperature Thermoelectric Power Generation. *Nano Lett.* **2015**, *15* (11), 7467–7472. https://doi.org/10.1021/acs.nanolett.5b03033.

(5) Ferrer, J.; Lambert, C. J.; García-Suárez, V. M.; Manrique, D. Z.; Visontai, D.; Oroszlany, L.; Rodríguez-Ferradás, R.; Grace, I.; Bailey, S. W. D.; Gillemot, K.; Sadeghi, H.; Algharagholy, L. A. GOLLUM: A next-Generation Simulation Tool for Electron, Thermal and Spin Transport. *New J. Phys.* **2014**, *16* (9), 093029. https://doi.org/10.1088/1367-2630/16/9/093029.

(6) Hoover, W. G. Canonical Dynamics: Equilibrium Phase-Space Distributions. *Phys. Rev. A* **1985**, *31* (3), 1695. https://doi.org/https://doi.org/10.1103/PhysRevA.31.1695.

(7) Nosé, S. A Unified Formulation of the Constant Temperature Molecular Dynamics Methods. *J. Chem. Phys.* **1984**, *81* (1), 511–519. https://doi.org/https://doi.org/10.1063/1.447334.

(8) Wang, J. J.; Gong, J.; McGaughey, A. J. H.; Segal, D. Simulations of Heat Transport in Single-Molecule Junctions: Investigations of the Thermal Diode Effect. *J. Chem. Phys.* **2022**, *157* (17), 174105. https://doi.org/https://doi.org/10.1063/5.0125714.

(9) Plimpton, S. Fast Parallel Algorithms for Short-Range Molecular Dynamics. *J. Comput. Phys.* **1995**, *117* (1), 1–19. https://doi.org/10.1006/jcph.1995.1039.

(10) Thompson, A. P.; Aktulga, H. M.; Berger, R.; Bolintineanu, D. S.; Brown, W. M.; Crozier, P. S.; In't Veld, P. J.; Kohlmeyer, A.; Moore, S. G.; Nguyen, T. D. LAMMPS-a Flexible Simulation Tool for Particle-Based Materials Modeling at the Atomic, Meso, and Continuum Scales. *Comput. Phys. Commun.* **2022**, *271*, 108171. https://doi.org/https://doi.org/10.1016/j.cpc.2021.108171.

(11) Grochola, G.; Russo, S. P.; Snook, I. K. On Fitting a Gold Embedded Atom Method Potential Using the Force Matching Method. *J. Chem. Phys.* **2005**, *123* (20), 204719. https://doi.org/https://doi.org/10.1063/1.2124667.

(12) Malde, A. K.; Zuo, L.; Breeze, M.; Stroet, M.; Poger, D.; Nair, P. C.; Oostenbrink, C.; Mark, A. E. An Automated Force Field Topology Builder (ATB) and Repository: Version 1.0. *J. Chem. Theory Comput.* **2011**, *7* (12), 4026–4037. https://doi.org/https://doi.org/10.1021/ct200196m.

(13) Pohjolainen, E.; Chen, X.; Malola, S.; Groenhof, G.; Hakkinen, H. A Unified AMBER-Compatible Molecular Mechanics Force Field for Thiolate-Protected Gold Nanoclusters. *J. Chem. Theory Comput.* **2016**, *12* (3), 1342–1350. https://doi.org/https://doi.org/10.1021/acs.jctc.5b01053.

(14) Mahaffy, R.; Bhatia, R.; Garrison, B. J. Diffusion of a Butanethiolate Molecule on a Au {111} Surface. *J. Phys. Chem. B* **1997**, *101* (5), 771–773. https://doi.org/https://doi.org/10.1021/jp962281w.
